# Functional Properties of Collagen Extracted from Catfish (*Silurus triostegus*) Waste

**DOI:** 10.3390/foods11050633

**Published:** 2022-02-22

**Authors:** Ayat A. Abbas, Khalida A. Shakir, Marie K. Walsh

**Affiliations:** 1 Biotechnology Research Center, University of AL-Nahrain, Baghdad 64074, Iraq; ayatadnan79@gmail.com; 2 Department of Food Science, College of Agricultural Engineering Science, University of Baghdad, Baghdad 10071, Iraq; dr_khalida55@yahoo.com; 3 Department of Nutrition, Dietetics, and Food Sciences, Utah State University, Logan, UT 84322-8700, USA

**Keywords:** catfish collagen, acid solubilized, pepsin solubilized

## Abstract

Collagen is used for a variety of biomedical and pharmaceutical uses, such as osteoarthritis-related pain management, hypertension, tissue engineering, and human implants, and is generally derived from porcine or bovine. Collagen from these animals has limitations due to the risk of disease transmission and religious constraints. Therefore, this study investigated the extraction of collagen from catfish (*Silurus triostegus*) waste. Acid-solubilized collagen and pepsin-solubilized collagen were extracted from catfish skin, fin, head, bone, and muscle. SDS-PAGE patterns of the extracted collagen showed that the protein molecular weights ranged from 97 to 200 kDa and skin, bone, and fin collagen consisted of 2 distinct α chains, which is typical of type 1 collagen. The proximate composition (moisture, protein, fat, and ash) and yield of the obtained extracts were determined. Skin collagen extracts were selected for further investigation due to the high collagen yield. The effects of the pH and salt concentration on solubility, and the denaturation temperature, FTIR spectra, reverse-phase HPLC, and SEM analysis were investigated to characterize the collagen samples. Based on the characterization of catfish skin collagen, this waste material has potential for use in the pharmaceutical and food industries.

## 1. Introduction

Catfish is a popular food, particularly on the continents of Africa and Asia. Collagen is a very well-known protein in vertebrates and comprises 30 percent of the total protein [1]. The word ‘collagen’ is derived from the Greek words ‘kolla’ and ‘genos’, meaning glue and shape, respectively. Collagen in the skin of cows and pigs is the most abundant collagen, but outbreaks of certain animal diseases, such as bovine spongiform encephalopathy and foot and mouth disease, have limited the use of animal collagen as these diseases are likely to be transmitted to humans [1]. Furthermore, Hindus do not eat cow-based products, and Muslims and Jews do not consume any food products linked to pigs [2]. Collagen has a wide variety of biomedical and pharmaceutical uses, such as osteoarthritis-related pain management, hypertension, tissue engineering, human implants, and angiogenic disease inhibition [1]. It is also used for drug delivery, skin replacements, expandable intra-arterial stents, and cell attachment substrates as a dermal filler [3]. Gelatin is a partly hydrolyzed type of collagen and is used in microencapsulation, food processing, and light-sensitive coatings in the food and packaging industries [3].

Due to its high availability, no chance of disease transmission, and no religious barriers, the best substitute for cow and pig collagen is fish collagen. The main differences between fish collagen and animal collagen are its high biological value, high essential amino acid content, and low hydroxyproline content [1]. There is growing interest in the purification of collagen from fish waste. Collagen from fish skin, bones, and fins has denaturation temperatures that are dependent on the indigenous water environment and are typically between 25 and 35 °C compared to mammalian collagen, which has a denaturation temperature of about 40 °C [1].

Fish processing discards, from unutilized and underutilized fish species, are promising sources for the extraction of fish collagen. Fish processing discards generally include skin, bones, scales, and fins. Hadfi and Sarbon [4] stated that in the fish processing industry, 20 to 80% of fish waste is generated depending on the level of processing and type of fish. Waste is generally dumped on land or hauled into the ocean. The disposal of these wastes also poses environmental problems for seafood processors.

Studies have investigated the extraction and characterization of collagen from different fish, including small-spotted catshark (*Scyliorhinus canicular*), rabbitfish (*Chimaera monstruosa*), lantern shark (*Etmopterus* spp.), catshark (*Galeus* spp.), catfish (*Ictalurus punctatus*), and the scales and fins of *Catla catla* and *Cirrhinus mrigala* [1]. Collagen extraction generally consists of preparation or washing, cleaning, and cutting of the fish samples. This is followed by chemical pretreatment with alkali to increase the efficacy of the extraction and to remove non-collagenous substances. For bones, cartilage, and scales, demineralization of the raw material with EDTA is performed to enhance the collagen extraction efficiency. Two primary extraction methods, acid and acid plus pepsin, are used to yield acid-soluble collagen (ASC) and pepsin-soluble collagen (PSC). Acetic acid is the most common acid used to extract collagen with a range of concentrations from 0.2 to 1 M, but a concentration of 0.5 M acetic acid is the most common [1]. Higher concentrations can degrade the proteins and reduce yields. The yields range from 0.4 to 40% depending on the collagen source and fish type [1]. The use of pepsin and acetic acid involves cleavage of the telopeptide regions of the triple helix, facilitating the leaching of collagen peptides into the solution and generally resulting in higher yields than the use of acetic acid alone [1].

This study was conducted to extract and classify ASC and PSC from the skin, fins, bone, head, and muscle of catfish, *Silurus triostegus*, which has not been reported in the literature. The extraction efficiency was determined, and skin collagen extracts were then characterized with respect to the molecular weight via SDS-PAGE, with solubility as a function of the pH and salt concentration, denaturation temperature, FTIR spectra, reverse phase high-performance liquid chromatography (HPLC), and morphology by SEM.

## 2. Materials and Methods

### 2.1. Materials

Catfish waste was collected from a local market in Baghdad and was transported in an ice box to the laboratory and stored at −18 °C. Pepsin (1:10,000) was purchased from Hi-Media Laboratories, India. Type I fish scale collagen was purchased from Scharlau Pure Chemicals, Spain. Unless specified otherwise, all chemicals used in the present work were of analytical grade.

### 2.2. Collagen Extraction

The skin and muscle were first cut into small pieces of about 0.5 cm in length, then pre-treated using 0.1 M NaOH at a sample:alkaline ratio of 1:8 (*w*/*v*) to remove non-collagenous proteins. The mixture was stirred for 6 h, with the solution removed and new solution added every 3 h. The de-proteinized skins were washed with distilled water until a pH of 7 was obtained, then defatted at a 1:10 (*w*/*v*) sample:alcohol ratio overnight with 10% butyl alcohol, and thoroughly washed with distilled water according to [4].

Collagen was extracted twice with acetic acid to obtain ASC using 10 volumes of 0.5 M acetic acid per gram of sample for 3 days according to [4]. The solution was centrifuged at 9000× *g* for 30 min at 4 °C after each extraction. The supernatant, or acid-soluble fraction, was subjected to a salting stage by adding NaCl to a final concentration of 2.5 M in the presence of 0.05 M tris (hydroxymethyl) aminomethan, pH 7.0. The precipitates were then collected by centrifugation at 9000× *g* for 30 min at 4 °C and dissolved in 0.5 M acetic acid. The sample was dialyzed against 0.1 M acetic acid in distilled water for 24 h. Lastly, this acid-soluble collagen sample was freeze dried using a VIRTIS sentry freeze-dryer (Virtis Company) and the dry samples were stored at −20 °C until further analysis.

For PSC, the deproteinized and defatted skins were treated by adding 0.1% (*w*/*v*) pepsin to 0.5 M acetic acid as described in [5,6]. Pepsin was added to cleave the telopeptide, a non-helical region increasing the collagen’s solubility. PSC was extracted twice for 3 days using 10 volumes of acetic acid:pepsin per gram of sample. Collected samples were heat treated at 80 °C for 5 min to inactivate the pepsin before lyophilizing the collagen as descried above.

For ASC and PSC from fins, bones, and head, after treatment with NaOH and washing with distilled water, an EDTA (0.5 M) solution was added containing 10 percent butyl alcohol (1:10 *w*/*v*) to decalcify and de-fat the samples. These samples were kept for 5 days in the solution. The samples were washed with water and then subjected to ASC and PSC treatment as described above for catfish skin and muscle. All collage samples were extracted 3 times and samples pooled.

### 2.3. Proximate Analysis of Samples

The soluble protein concentration was calculated using the biuret method [7] using a UV-VIS-spectrophotometer (Selecta, Barcelona, Spain) at 540 nm. The tissue total protein content was determined by the Kjeldahl method [8]. The moisture content was determined by the reference method [9]. The total content of lipids and ash in fish tissue was determined according to a standard method [10]. The yields of ASCs and PSCs were determined based on the dry weight of pretreated material using the following equation: yield (g/100 g) = (weight of lyophilized collagen)/(weight of initial dry fish pretreated byproduct) × 100 [4,11]. All measurements were performed in triplicate and the values are presented as means and standard deviations as calculated in Microsoft Excel 2016, Office 365. Significance was determined at the alpha level of 0.05.

### 2.4. Collagen Yield and Recovery

Collagen yield and recovery were determined by measuring the hydroxyproline content since all the hydroxyproline in the samples was assumed to be from collagen. The hydroxyproline content was calculated by the colorimetric method as described by [12,13] after the material was hydrolyzed in 6 MHCl for 6 h at 105 °C. Hydrolyzed samples (2 mL) were mixed with a buffered chloramine-T reagent (1.5 mL, pH 6.5), and oxidation was allowed to proceed for 25 min at 20 °C. Ehrilich’s aldehyde reagent (1.5 mL) was added to each sample, mixed, and the chromophore was generated by incubating the sample at 65 °C for 20 min [12]. The absorbance was measured with a spectrometer at 550 nm (Selecta, Barcelona, Spain). The recovery based on the hydroxyproline content was calculated according to the following equation: recovery (%) = hydroxyproline in lyophilized purified collagen/hydroxyproline in dry tissue × 100. All measurements were performed in triplicate.

### 2.5. Influence of pH on Collagen Solubility

The collagen sample (1 g dry weight) was dissolved in 8 mL of 0.5 M acetic acid and transferred to a 50-mL centrifuge tube. To obtain a final pH that ranged from 1 to 12, the pH was changed using 6 M of either NaOH or HCl. Samples were then brought to 10 mL with deionized water. Samples were centrifuged (Selecta, Barcelona, Spain) at 6000× *g* at 4 °C for 30 min. Using the Biuret method [7], the protein content in the supernatant was calculated. The estimation of the relative solubility was calculated based on the value of the solubility at each pH value using the following equation: relative solubility (%) = protein content of supernatant at current pH/ total protein content in sample × 100. All measurements were performed in triplicate. The values are presented as means and standard deviations as calculated in Microsoft Excel 2016, Office 365.

### 2.6. Influence of NaCl on Collagen Solubility

Collagen, 1 g dry weight, was mixed with 5 mL of 0.5 M acetic acid containing different NaCl concentrations (*w*/*v* from 0 to 12%). The mixture was stirred continuously for 30 min at 4 °C and then centrifuged for 15 min at 6000× *g* at 4 °C using a centrifuge (Selecta, Barcelona, Spain). The concentration of protein in the supernatant was measured by the Biuret method [7]. The relative solubility at each NaCl concentration was measured and the relative solubility was calculated according to the equation: relative solubility (%) = protein content of supernatant/total protein content in sample × 100. All measurements were performed in triplicate. The values are presented as means and standard deviations as calculated in Microsoft Excel 2016, Office 365.

### 2.7. Temperature-Induced Change in Viscosity

The denaturation temperature of collagen was calculated based on temperature-induced viscosity changes using an Ostwald viscometer [6,14,15]. Solutions of 0.1% (*w*/*v*) collagen (0.997 g/mL) were prepared at 10 °C with 0.1 M acetic acid. The samples were incubated for 30 min at 10 °C. Then, the temperature was steadily increased to 15, 20, 25, 35, 35, 40, 45, 50, and 55 °C and the samples were incubated at each temperature for 30 min. All measurements were performed in triplicate. The fractional viscosity was measured for each temperature as follows:

Sample Viscosity/Water viscosity = P_2_t_2_/P_1_t_1_

t_1_: Time for water to pass.

P_1_: Water density.

t_2_: Time for sample to pass.

P_2_^:^ Sample density.

Fractional viscosity = (measured viscosity at each temperature − minimum viscosity)/(maximum viscosity − minimum viscosity).

The denaturation temperature (Td) is the temperature at which the fractional viscosity value is half (0.5) of the highest viscosity value. The values are presented as means and standard deviations as calculated in Microsoft Excel 2016, Office 365.

### 2.8. Protein Molecular Weight Determination by SDS-PAGE

SDS-PAGE was performed using the discontinuous Tris- HCl/glycine buffer system with a 10 percent resolving gel with 5 percent stacking gel according to [16]. The collagen sample was dissolved in sample buffer with 10% β mercaptoethanol (0.5 M Tris HCl, pH 6.8, containing 2% SDS and 25% glycerol) to achieve a final collagen concentration of 1 mg/mL and then heated for 3 min and 10 μL were loaded into the gel. The gel was stained with 0.25% Coomasie Brilliant Blue R- 250 solution for 30 min and then with 7.5% acetic acid containing 5% methanol solution until the bands were clear.

### 2.9. FTIR Spectra and SEM Analysis

FTIR was performed using KBr pellets obtained from discs containing 200 mg of potassium bromide (KBr) containing 2 mg of dry collagen at the Ministry of Industry and Minerals (Baghdad, Iraq). The Shimadzu (Tokyo, Japan) model instrument used has a wavelength of 400–4000 cm^−1^ and FTIR measurements were conducted as described by [4].

Structural examination was performed by scanning electron microscopy (SEM) of the extracted dry collagen. The sample was gold coated with an automated fine ion coater (JEOL JFC-1600) and the structure was examined with a 20 kV scanning electron microscope (TESCAN, VEGA III). This was conducted at the Ministry of Science and Technology (Baghdad, Iraq).

### 2.10. High-Pressure Liquid Chromatography

For chromatographic separation, a high-performance liquid chromatography (HPLC) method using a HPLC system (Jasco AS-950-10 injector, a Jasco PU-980 pump, and a Shimadzu ERC- 7515A diode array detector) with a flow rate of 0.8 mL/min was used. The column was a Vydac reverse phase C18 (4.6 × 250 mm, 4.6 mm i.d., 5.0 μm). For the chromatographic separation, an isocratic gradient with a mobile phase consisting of 150 mM of phosphate buffer, pH 7.0 with 25% HPLC-grade acetonitrile at room temperature was used. To eliminate carry-over contamination, the auto-sampler syringe and the injection valve were successively washed with methanol/water (70/30; *v*/*v*) between samples. In total, 5 μg of sample were assayed and samples were detected at a wavelength of 214 nm. Sample preparation was performed according to [17].

## 3. Results and Discussion

### 3.1. Proximate Analysis

Table 1 shows the composition of catfish *Silurus triostegus* muscle, head, fins, bone, and skin. The moisture content in the experimental samples varied from 68 to 72.5% and there were no significant differences between the skin, bone, head, and fins. However, the moisture content of the muscle sample was significantly higher (72.50%) than others. These findings are in accordance with Sato et al. [18], who stated that the moisture content of fish species muscles ranged between 54 and 80%. Boran and Karacam [19] found that the moisture content of shad fish (edible part) ranged from 57–68% and from 65 to 73.40% for horse mackerel, which is close to our findings. In general, fish muscle has a high capacity to retain water relative to other flesh, such as beef, before slaughter or manufacturing [20]. The catfish skin moisture content was 68.5%, which is less than that of the brown backed toadfish (73.40%) [3].

The ash content of the experimental samples varied significantly, with values ranging from 0.345 to 0.660%. These values are close to the value of 0.42% reported by Aum-El-Basher et al. [21] for Oriental sole (*Brachirus orientalis*), and lower than that obtained (1.45%) for 4 tropical species (*Clarias gariepinus*, *Selar crumenophthalmus*, *Scomber scrombus*, *and Pseudotolithus*) [22]. Matmaroh et al. [23] stated that the ash content in the scales of spotted golden goatfish was 0.442%. The variation in the ash content could be due to variations in the fish’s age, sex, and environment [24]. Fish scales have a high ash content due to the high amounts of calcium salts Ca_5_(PO_4_)_3_OH.

The fat content varied significantly from 0.620 to 3.85%. The percentage of catfish skin fat (2.50%) in this study was similar to *Brachirus orientalis* fish skin (2.58%) and to *Pangasianodon gigas* skin (2.69%) [21]. However, it was lower than rainbow trout skin (13.12%) [25] and slightly higher than the percentage of fat in Nile tilapia skin (1.1%) [26].

The protein content is shown in Table 1 and the protein contents of catfish skin and muscle were significantly higher than that of fin, bone, and head. High protein contents (23.92%) in the skin of catfish may be an indicator that it is a good source of collagen. Muyonga et al. [27] stated that Nile perch skin contained 20 to 22% protein, which is similar to the results of this study. However, the protein percentage of Oriental sole (*Brachirus orientalis*) skin was 29.27% [21]. Abbey et al. [28] reported that the protein content of raw tuna muscle was 29% while Thitipramote and Rawkdkuen [29] found that striped catfish skin had a lower protein content of 20.24%. The composition of catfish skin reported here is comparable to that reported by Xu et al. [5]. The different chemical compositions of fish are influenced by biological factors, such as species [30].

### 3.2. Collagen Yield and Recovery

Table 2 shows the collagen yield, protein recovery, and hydroxyproline content of ASC and PSC extracted from catfish skin, bone, muscle, fin, and head. The collagen yield from different sources shows variation, and this is similar to Muralidharan et al. [6], who stated that the collagen yield from various parts of leather jacket (*Odonus niger)* showed variation. The protein content values were 0.37, 0.20, 0.57, 0.32, and 0.13 mg/mL for ASC from skin, bone, muscle, fin, and head, respectively. For PSC, the values were 0.52, 0.33, 0.60, 0.44, and 0.25 mg/mL, respectively, for the same samples. The variation in the collagen yield was similar to Normah and Maidzatul Afiquah [30], who found that the collagen yield from a mixture of bone, scale, and skin of sin croaker (*Johniecop sina*) was 2.74 and 3.35% as a function of 3 and 5 days of extraction using 0.5 M acetic acid. The variation in the yield could be due to the extraction conditions. In all samples, the PSC yield and recovery were greater than ASC. This could be attributed to the function of pepsin, which cleaves the covalent cross-links at the telopeptide, resulting in increased collagen solubility [6,15,31].

As a means to measure the amount of collagen in a specific tissue, hydroxyproline measurement was used considering that all the hydroxyproline in samples was from collagen [32]. The results in Table 2 show that the hydroproline values in skin, bone, muscle, fin, and head ASC samples were 4.92, 4.10, 3.78, 3.50, and 1.99 µg/mL, respectively, while the recovery values were 69.7%, 63.06%, 52.1%, 51.5%, and 47.2%, respectively, for the same samples. For PSC, the hdyroxyproline values were 6.14, 5.74, 5.39, 5.53, and 3.15 µg/mL, respectively. The recovery values were 86.93, 61.2, 74.7, 81.5, and 72.6% for the same samples, respectively. These values were different from an earlier study, which found that the collagen yield of the bones, skin, scales, and fins of marine fish ranged from 30% to 51% [33]. Muralidharan et al. [6] reported that the collagen yield (based on hydroxyproline recovery) of leather jacket skin, bone, and muscle varied from 46.48% to 70.94%. The variation in the hydroxyproline recovery and yield could be attributed to differences in the extraction conditions, fish species, and structural differences in the collagen [34,35].

The collagen yield values for ASC ranged from 0.10 to 2.6% while for PSC, they ranged from 0.58 to 8.24%. The highest values in both ASC and PSC were observed for the skin samples. As previously reported, the collagen yield of big eye snapper bone (acidic extraction) was 1.6% and that of spotted goatfish scales and silver carp was 0.46% and 1.45%, respectively [23,32]. Duan et al. [36] found yields of 1.35 and 1.06% collagen in bone samples and the concentration of acetic acid affected the extractability of the collagen. Hamdan and Sarbon [37] and Veeruaj et al. [38] found the ASC yield was higher than PSC, and they attributed their findings to the low efficiency of pepsin or the insufficient duration of the extraction process. Jonggareonrak et al. [39] and Hamdan and Sarbon [37] suggested that restricted digestion of pepsin meant that the cross-linked molecules at the telopeptide area were cleaved without complete destriuction of the structure of collagen’s triple helix, which contributed to the lower yield of the PSC samples. All these findings were lower than those reported by Slimane and Sadok [15], who found that the ASC and PSC yields of cartilaginous fish were 23.07% and 35.27% on a dry weight, respectively.

### 3.3. SDS-PAGE of Collagen Extracts

**Figure 1:**
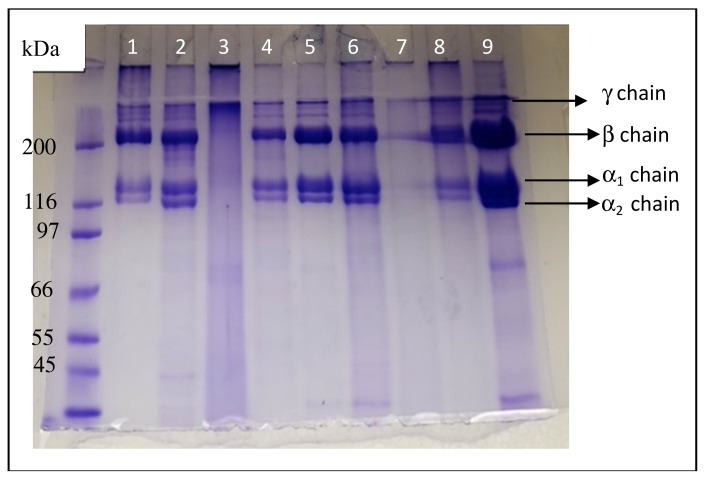
SDS-PAGE analysis of acid-solubilized collagen (ASC) and pepsin-solubilized collagen (PSC) from catfish (1) PSC skin, (2) PSC fin, (3) PSC muscle, (4) PSC head, (5) ASC head, (6) ASC bone, (7) ASC muscle (8) ASC fin, and (9) ASC skin.

Figure 1 shows the SDS-PAGE of ASC and PSC from catfish skin, bone, fin, muscle, and head. A marker with a molecular weight was used to estimate the molecular weights of the experimental collagen protein bands. The electrophoretic profiles also showed that there were no significant differences between the experimental collagen profiles for the same tissue derived from ASC or PSC, and similar protein patterns were observed except for muscle ASC and PSC. The muscle samples showed blurry protein bands, which may have been due to the extraction method. The electrophoretic patterns revealed that all samples had fractions with molecular weights ranging from 66 kDa to greater than 200 kD and consisted of α chains (α_1_ and α_2_) and their dimer a β chain, except the muscle samples. These patterns were similar to those of PSC from grass carp [40]. These findings were also similar to those reported by [41,42,43], who found that the molecular weight of α chains from marine fish skin was about 120 and 110 KD, respectively.

All collagen, except from muscle, comprised two α chains and β chains. The β chain molecular weight was about 200 KDa and the α chain molecular weights were similar to and slightly larger than 116 KDa. The presence of a presumed γ chain that did not enter the resolving gel indicates that the three chains of collagen were intra-molecularly cross-linked while the presence of the β chain indicates that the collagen contains inter-molecular interactions.

Additionally, the skin, head, bone, and fin collagen consisted of two distinct α chains, which is typical of type 1 collagen, consisting of α chains (α_1_ and α_2_) and their dimer, a β chain. The isolated collagen may be type I, which was previously observed by Xu at al. [5] for catfish skin and Slimane and Sadok [15] for *Mustelus mustelus* skin. Muyonga et al. [27] found that the molecular weight of the α fractions from type I skin collagen of Nile perch was 120 and 115 kDa, respectively. Skierka and Sudowska [44] found that the molecular weight of the α fractions of Baltic cod was below 115 kD. Type II collagen is composed of three α_1_ chains, so, due to the presence of α_1_ and α_2_ chains, catfish skin collagen is presumably type I collagen.

### 3.4. Effect of pH and NaCl on Collagen Solubility

**Figure 2:**
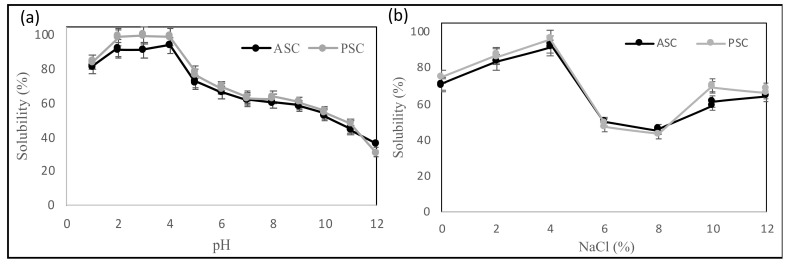
Relative solubility (%) of acid (ASC)- and pepsin (PSC)-solubilized catfish skin collagen as affected by different (**a**) pH and (**b**) NaCl concentrations.

Due to the high yield and recovery of collagen from the skin, skin collagen extracts were chosen for further investigation. Figure 2a shows the solubility of catfish skin collagen (ASC and PSC) as influenced by various pH values. At different pH values, both collagens displayed a similar solubility pattern. At very acidic pH values, high solubility was observed (pH 2 to 4). At pH 4, the maximum solubility was found to be 91.32% and 99.63% for ASC and PSC, respectively.

Similar findings were recorded for snakehead collagen (*Ophiocephalus argus*), brownstripe red snapper (*Lutjanus vitta*), and bigeye snapper (*Priacanthus macracanthus*), with higher solubility at pH values ranging from 1 to 4 [39,45]. Tan and Chang [43] showed that both ASC and PSC from catfish skin had high solubility at pH values less than 6 while only 20% solubility at pH values greater than 6, which is different from the solubility pattern observed here. This may be due to the different species of catfish used. The solubility of collagen is also affected by its conformations and molecular characteristics [32].

The effect of different concentrations of NaCl on the solubility of catfish skin collagen is shown in Figure 2b. The solubility of collagen was NaCl concentration dependent: the solubility increased as the NaCl concentration increased from 0 to 4% (*w*/*v*), then decreased sharply at 5% NaCl, and stayed low until a slight increase in solubility at 10 and 12% NaCl. At the 0 concentration of NaCl, the solubilities were 71% and 75% for ASC and PSC, respectively. This finding is consistent with previous studies, where, at increased NaCl concentrations, the solubility of different collagens decreased [32,39,46,47]. Tan and Chang [43] showed a similar salt-dependent solubility for catfish skin collagen, which showed high solubility at salt concentrations ranging from 0 to 3% but only 40% solubility at salt concentrations greater than 4%.

### 3.5. Temperature-Induced Change in Viscosity

**Figure 3:**
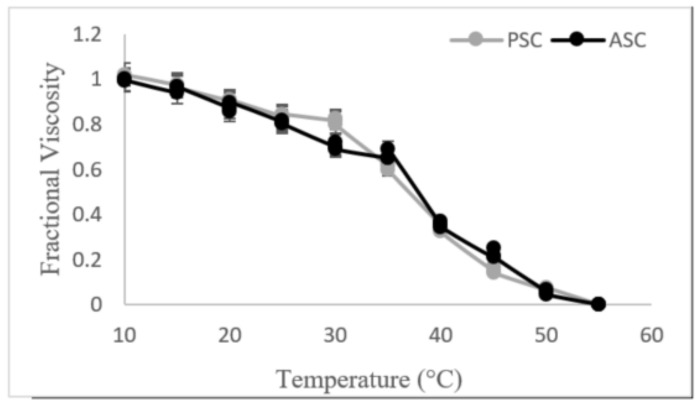
Variations in the fractional viscosity of acid-soluble collagen (ASC) and pepsin-soluble collagen (PSC) from catfish skin as a function of temperature.

The temperature of denaturation (Td) of ASC and PSC from catfish skin was calculated using the temperature-mediated change in viscosity, as shown in Figure 3. The Td of catfish skin ASC and PSC was found to be 35 °C for each, which is lower than the Td of porcine skin collagen (39 °C) and that of *Labeo rohita* and *Labeo catla* fish (36.5 °C) [1,14]. Collagen derived from marine fish, such as the common mackerel, showed a Td of 26.9 °C and chum salmon showed a Td of 20.6 °C [48]. The difference in Td is mainly determined by the temperature of the environment in which the fish resides [14].

### 3.6. FTIR Analysis

Fourier transform infrared technology was applied to identify the vibrational modes of bands and individual chemical groups in the extracted collagens. Spectra were acquired at a resolution of 4 cm^−1^ and the measurement range was 4000–600 cm^−1^ (mid-IR region) [49]. Amide A commonly appears in the range of 3300 to 3440 cm^−1^”in type I collagen, as shown in Table 3, and is involved in N-H stretching coupled with hydrogen bonds [1]. The collagen’s FTIR spectra shown in Table 3 provides the amide A values of ASC and PSC, which were within the range reported for type I collagen. The absorption peak of amide B is related to asymmetrical stretching of CH_2_ [27], and all samples had similar values. The changes in the wavenumber and amplitude of amides A and B found in ASC and PSC may be due to the different extraction methods.

The amide I peak is associated with C=O stretching and is a marker of the secondary structure in proteins [50] while the amide II peak results from N-H bending coupled with C-N stretching [49]. Amide III is associated with an N-H bend. All samples contained peaks within the expected ranges for each amide. These results are similar to data reported by Hadfi and Sarbon [4] and indicate that the functional groups in ASC and PSC were not damaged by the acidic conditions and pepsin used in the extraction process.

### 3.7. High-Performance Chromatography

**Figure 4:**
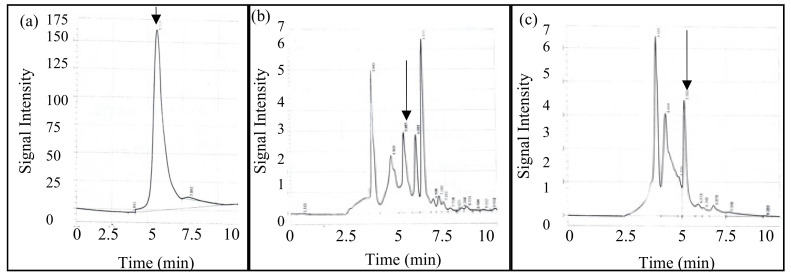
High-performance liquid chromatography of collagen (**a**) type I collagen; (**b**) pepsin-solubilized catfish skin; (**c**) acid-solubilized catfish skin. The arrow indicates a retention time of 5.5 min.

HPLC chromatograms of type I collagen, PSC, and ASC of catfish skin are shown in Figure 4. The protein samples were separated by reverse phase chromatography, resulting in the least hydrophobic proteins/peptides being eluted before more hydrophobic proteins/peptides. The type I collagen standard (Figure 4a) showed 1 major peak at a retention time of 5.5 min, and this constituted 96.4% of the total area obtained by all peaks. The ACS sample (Figure 4c) also contained a peak at 5.5 min that corresponded to 16.5% of the total area obtained by all peaks. Additionally, this sample showed peaks at times of 4 and 4.5 min, which corresponded to 35 and 34% of the total area, respectively.

The PSC sample (Figure 4b) also contained a peak at 5.5 min that corresponded to 17.8% of the total area of all peaks. This sample also contained peaks at times of 4 and 4.5 min as in the ACS sample, which corresponded to 17.3 and 19% of the total area. Additionally, the PSC sample showed peaks at 6 and 6.4 min, which corresponded to 10 and 20.2% of the total area of all peaks. This sample also contained multiple peaks that eluted after 6.4 min and are presumed to be hydrophobic peptides. Each sample showed a peak with the same retention time as type I collagen, but the PSC sample contained two additional peaks compared to the ACS sample, presumably due to pepsin hydrolysis. This separation was done based on the hydrophobicity of the proteins/peptides in the sample, not by size, so presumably the peak at the 5.5 min retention time may have contained β, α_1_, and α_2_ chains and the other protein/peptide peaks arose from the solubilization method used.

### 3.8. Scanning Electron Microscopy (SEM)

**Figure 5:**
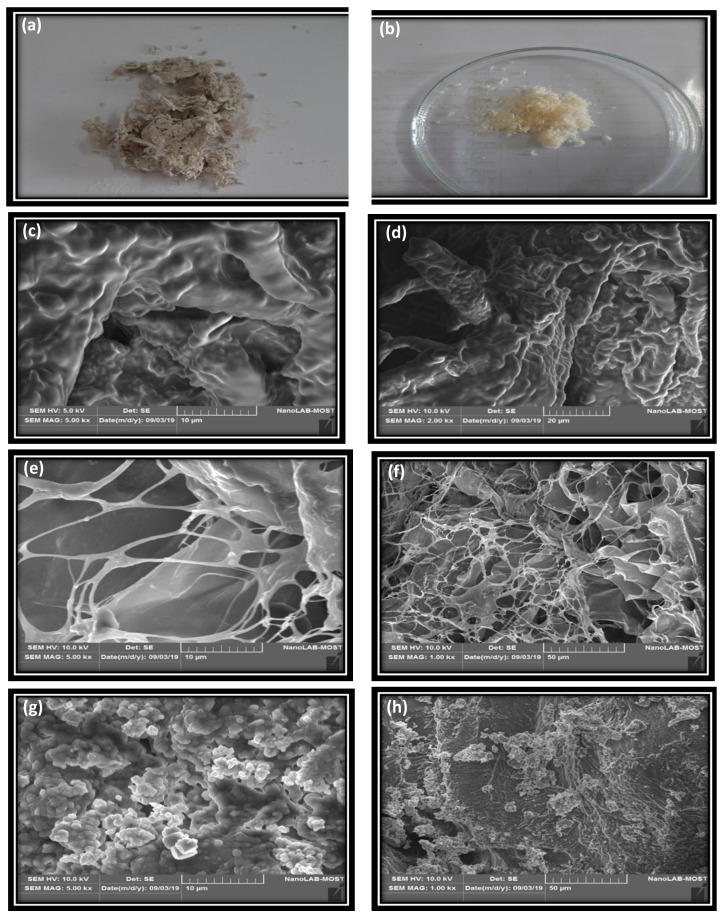
Visual representation of (**a**) ASC, (**b**) PSC, and microscopic structures of (**c**) SEM standard type I collagen at 10 mm, (**d**) SEM standard type I collagen at 20 μm, (**e**) ASC at 10 μm, (**f**) ACS at 50 μm, (**g**) PSC at 10 μm, (**h**) PSC at 50 μm.

A change in the structural morphology of fish skin collagen was observed by SEM images, as shown in Figure 5. The morphology of fish skin collagen varied among the samples, as shown in the visual photo and the SEM pictures. As shown in Figure 5a,b, the ASC and PSC appeared as a soft white spongey material. The SEM analysis of type I collagen, ASC, and PSC is illustrated in Figure 5c–h. The surface morphology of type I collagen (Figure 5c,d) shows a rough surface with some fibrous content. ACS (Figure 5e,f) shows highly fibrous structures while PSC shows more globular structures. Vairamani [51] analyzed the acid-soluble and pepsin-soluble collagen of the outer skin of spineless cuttlefish *Sepiella inermis* via SEM and both showed fibrous-type structures. Hadfi and Sarbon [4] analyzed silver catfish skin collagen via SEM and showed that the acetic-acid-extracted collagen was highly fibrous. Presumably, the extraction and sample preparation methods influence the structure of the final collagen sample.

## 4. Conclusions

This study investigated the extraction of collagen from catfish (*Silurus triostegus*) skin, fin, head, bone, and muscle using acetic acid (ASC) and pepsin with acetic acid (PSC) extraction methods. For each sample, the PSC yield was higher than the ASC yield and, specifically, the collagen yields from the skin were the highest. Skin, fin, head, and bone collagen comprised α_1_, α_2_, and β chain proteins, which is typical of type I collagen. Skin collagen was further characterized and both skin ASC and PSC collagen showed high solubility at pH values of 2 to 4 and with 0 to 4% sodium chloride. Reverse phase HPLC analysis showed multiple protein peaks in the PSC samples compared to the ASC and type I collagen standard samples, presumably due to pepsin hydrolysis. SEM analysis of ACS showed fibrous structures while PSC contained more globular structures. The denaturation temperature of catfish collagen was determined to be 35 °C, which is lower than porcine collagen and may limit its use in biomedical applications, but it could be a replacement for porcine collagen in the food industry.

## Figures and Tables

**Table 1:** Composition of catfish samples. Within columns, values with different letter superscripts are significantly different.

Sample	% Protein	% Moisture	% Fat	% Ash
Muscle	20.64 ± 0.35 ^a^	72.50 ± 0.50 ^a^	3.85 ± 0.05 ^a^	0.345 ± 0.03 ^a^
Head	15.87 ± 0.20 ^b^	68.00 ± 1.00 ^b^	2.04 ± 0.0 ^b^	0.210 ± 0.0 ^b^
Fin	18.15 ± 0.33 ^b^	69.00 ± 1.00 ^b^	0.62 ± 0.0 ^c^	0.470 ± 0.0 ^c^
Bone	19.93 ± 0.42 ^b^	68.50 ± 0.50 ^b^	1.82 ± 0.03 ^d^	0.580 ± 0.0 ^d^
Skin	23.92 ± 0.46 ^b^	69.50 ± 0.50 ^b^	2.50 ± 0.30 ^e^	0.660 ± 0.02 ^e^

**Table 2:** Protein recovery, hydroxyproline content, recovery, and yield of acid-soluble collagen (ASC) and pepsin-soluble collagen (PSC) from catfish samples.

Sample		Skin	Bone	Muscle	Fin	Head
	Protein (mg/mL)	0.37 ± 0.02	0.20 ± 0.00	0.57 ± 0.03	0.32 ± 0.03	0.13 ± 0.00
ASC	Hydroxyproline (µg/mL)	4.92 ±0.10	4.10 ± 0.00	3.78 ± 0.02	3.5 ± 0.00	1.99 ± 0.00
	Recovery (%)	69.7 ±1.33	63.06 ± 1.4	52.1 ± 1.43	51.5 ± 1.47	47.2 ± 0.70
	Yield (%)	2.6 ± 0.014	0.28 ± 0.02	0.56 ± 0.03	0.28 ± 0.03	0.10 ± 0.00
	Protein (mg/mL)	0.52 ± 0.00	0.33 ± 0.03	0.60 ± 0.03	0.44 ± 0.03	0.25 ± 0.14
PSC	Hydroxyproline (µg/mL)	6.14 ± 0.01	5.74 ± 0.28	5.39 ± 0.01	5.53 ±0.28	3.15 ± 1.40
	Recovery (%)	86.9 ± 1.43	61.2 ±1.43	74.7 ± 0.00	81.5 ± 0.01	72.6 ± 1.40
	Yield (%)	8.24 ±0.01	0.82 ± 0.03	0.74 ± 0.03	0.58 ± 0.06	0.94 ± 0.00

**Table 3:** Functional groups obtained from FTIR of acid-solubilized collagen (ASC) and pepsin-solubilized collagen (PSC) from catfish skin and type I collagen standard.

	Wavelength (cm^−1^)				
Sample	Amide 1	Amide II	Amide III	Amide-A	Amide-B
ACS	17,070.00	1537.26	1300.02	3425.58	2985.81
PSC	1699.29	1558.48	1253.73	3421.72	2927.94
Type I standard	1680.64	1507.05	1298.62	3298.28	2985.81
Average values ^1^	1620–1800	1590–1650	1200–1400	3300–3400	
Signal source ^1^	C=O stretch	C-N stretch and N-H bending	N-H bend	N-H stretch H bonding	

^1^ Average values and signal source in type I collagen as reported in Jafari [1].

## Data Availability

Not applicable.
